# COVID-19-specific adult basic life support guideline strategies for chiropractors and other healthcare providers to maximize the safety and efficacy of resuscitation: a commentary

**DOI:** 10.1186/s12998-023-00488-y

**Published:** 2023-06-05

**Authors:** Chun-Cheung Woo

**Affiliations:** CC Woo Chiropractic Clinic, Chatswood, NSW 2057 Australia

**Keywords:** COVID-19, SARS-CoV-2, Basic life support, Cardiopulmonary resuscitation, Aerosol transmission, Guidelines, Chiropractic, Healthcare providers

## Abstract

**Background:**

The emergence of an unprecedented novel severe acute respiratory syndrome coronavirus-2 (SARS-C0V-2), which causes the coronavirus disease 2019 (COVID-19) pandemic, has created new scenarios in basic life support (BLS) management. According to current evidence, SARS-CoV-2 can be transmitted airborne in aerosol particles during resuscitation. Research evidence found an alarming global increase in out-of-hospital cardiac arrests during the COVID-19 pandemic. Healthcare providers are legally obliged to respond to cardiac arrest as soon as possible. Chiropractors will likely encounter potential exercise-related and non-exercise-related cardiac emergencies at some point in their professional lives. They have a duty of care to respond to emergencies such as cardiac arrest. Chiropractors are increasingly involved in providing care, including emergency care, for athletes and spectators at sporting events. Also, exercise-related cardiac arrest in adult patients may occur during exercise testing or rehabilitation with exercise prescriptions in chiropractic and other healthcare settings. Little is known about the COVID-19 BLS guidelines for chiropractors. Knowledge of the current COVID-19-specific adult BLS guidelines is essential to developing an emergency response plan for the on-field and sideline management of exercise-related cardiac arrest and non-athletic, non-exercise-related cardiac arrest.

**Main text:**

Seven peer-reviewed articles on the COVID-19-specific BLS guidelines, including two updates, were reviewed for this commentary. Responding to the COVID-19 pandemic, the national and international resuscitation organizations recommended interim COVID-19-specific BLS guidelines with precaution, resuscitation, and education strategies. BLS safety is paramount. A precautionary approach with the bare minimum of appropriate personal protective equipment for resuscitation is recommended. There was disagreement among the COVID-19 BLS guidelines on the level of personal protective equipment. All healthcare professionals should also undergo self-directed BLS e-learning and virtual skill e-training. The summarized COVID-19-specific adult BLS guideline strategies and protocols are tabled, respectively.

**Conclusions:**

This commentary provides a practical overview and highlights current evidence-based intervention strategies of the COVID-19-specific adult BLS guidelines that may help chiropractors and other healthcare providers reduce BLS-related exposures to SARS-CoV-2 and the risks of SARS-CoV-2 transmission and maximize the efficacy of resuscitation. This study is relevant to and impacts future COVID-19-related research in areas such as infection prevention and control.

## Background

The emergence of an unprecedented novel severe acute respiratory syndrome coronavirus-2 (SARS-C0V-2), which causes the coronavirus disease 2019 (COVID-19) pandemic, has created new scenarios in basic life support (BLS) management. SARS-CoV-2 can be transmitted airborne in aerosol particles, according to recent evidence [1]. According to the American Heart Association 2020 Interim COVID-19 Guidance, SARS-CoV2 is highly transmissible during resuscitation and has a high morbidity and mortality rate, complicating the emerging response to out-of-hospital cardiac arrest (OHCA) [2].

Cardiac arrest is a time-critical, life-threatening emergency requiring prompt, urgent, and appropriate interventions. The biggest challenge during cardiopulmonary resuscitation (CPR) is to ensure the best possible chance of survival for an OHCA patient without compromising the safety of the healthcare rescuers [2–5]. According to the Australasian College of Emergency Medicine, it is important to balance the appropriateness of resuscitation against the risk of infection to manage cardiac arrest during the COVID-19 pandemic [3]. There are risks and concerns of SARS-CoV-2 aerosol transmission during AGPs, including chest compressions, defibrillation, bag-valve-mask (BVM) ventilation, and positive-pressure ventilation [5]. Considering the rapid spread of the Omicron variant of concern for SARS-CoV-2, the 2021 World Health Organization (WHO) recommends the appropriate use of a respirator for aerosol-generating procedures (AGPs) along with other personal protective equipment (PPE) by healthcare providers providing care in any setting to patients with suspected or confirmed COVID-19 [6].

A recent systematic review and meta-analysis found an alarming global increase in OHCA during COVID-19 [7]. The SARS-CoV-2-induced systemic inflammation could lead to cardiovascular complications such as acute coronary syndrome and myocarditis. Little is known about the incidence of OHCA in chiropractic and other healthcare settings before and during the COVID-19 pandemic. Before the COVID-19 pandemic, a 5 years cross-sectional study found that 36% of general medical practice was involved in OHCA, and 13% was involved in more than one OHCA [8]. A recent study from the Swedish Registry of Cardiopulmonary Resuscitation from 2011 to 2014 and 2016 to 2018 found 635 cases of exercise-related OHCA outside of the home [9].

Chiropractors will likely encounter potential exercise-related and non-exercise-related cardiac emergencies at some point in their professional lives. They have a duty of care to respond to emergencies such as cardiac arrest. Like other healthcare providers, chiropractors are increasingly involved in providing care at sporting events. Because sports chiropractors can be called on to provide BLS for athletes and spectators, knowledge of the current COVID-19 BLS guidelines is essential to developing an emergency response plan for the on-field and sideline management of exercise-related cardiac arrest and non-athletic, non-exercise-related cardiac arrest. Moreover, exercise-related cardiac arrest may occur during exercise testing or rehabilitation with exercise prescriptions for patients in chiropractic clinical settings.

Responding to the COVID-19 pandemic, the national and international resuscitation organizations recommended interim COVID-19-specific BLS guidelines [2–5, 10–12] with precaution, resuscitation, and education strategies based on expert opinion and consensus. Little is known about the COVID-19 BLS guidelines for chiropractors. This commentary will discuss the current evidence of CPR-associated SARS-CoV-2 transmission and the COVID-19-specific adult BLS guidelines for chiropractors and other healthcare providers, with guideline strategies in Table 1 and guideline protocols in Table 2, respectively.

**Table 1:** Summary of current intervention strategies of the COVID-19-specific adult BLS guidelines for healthcare providers to maximize the safety and efficacy of resuscitation

Key intervention strategies of the COVID-19-specific adult BLS guidelines and updates	
*Precaution strategies to reduce healthcare providers’ risks from SARS-CoV-2*	*Resuscitation strategies to reduce healthcare providers’ exposures to SARS-CoV-2 and provide timely care*
• Consider resuscitation appropriateness [2, 3, 11] and risk assessment [2, 10]	• Only look at the chest rise and fall to check breathing [2, 10, 11]
• Don appropriate PPE for AGPs before CPR [2, 3, 5, 10, 11]	• Cover the patient’s mouth and nose with a barrier device such as a surgical mask, or equivalent [3, 12,10-]
• Limit personnel to only rescuers [2, 10]	• Prioritize defibrillation when indicated [2-5, 12,10-]
• Limit unprotected rescuers’ exposure to AGPs, and they should be immediately excused from the CPR room or area [5, 12]	• Prioritize oxygenation and ventilation [2, 10]
• Relieve initial resuscitation personnel with providers wearing appropriate PPE [12]	• Initiate chest compression [3, 11] without delay or interruption while wearing appropriate PPE [5]
• Doff all PPE safely, avoiding self-contamination after CPR [3]	• Use a mechanical chest compression device if available and trained [2, 3, 5, 11, 12]
• Clean, disinfect, or dispose of all the used equipment and nearby surfaces according to local guidelines after resuscitation [3]	• Pause chest compressions during ventilation to minimize the risk of aerosol [2, 3, 10]
	• Ventilate with a BVM-HEPA filter using two hands with a tight seal to provide rescue breathing if required [2, 10, 12]
*Education strategies for healthcare providers to minimize the potential risks of SARS-CoV-2 transmission*	*Specific additional resuscitation strategy updates include*
• Undergo self-directed web-based e-learning and virtual skill e-training [10]	• Defibrillate as soon as possible if healthcare providers are wearing appropriate PPE for AGPs [5]
• Learn to self-protect against CPR equipment and procedure-related infections [10]	• Prioritize chest compressions and defibrillation when indicated [5, 12]
• Learn to use CPR-related equipment or procedures [10]	• Maximize the chest compression fraction and pause to intubate only if necessary [5, 12]
• Learn how to perform donning and doffing PPE [3, 10, 11]	• Securely attach a HEPA filter to any ventilation device [5, 12]
• Practice CPR procedures with consideration for using PPE [10]	• Consider passive oxygenation overlaid with a surgical mask for agonal breathing until HEPA-filtered ventilation can be provided [5, 12]

Compiled and modified based on COVID-19-specific BLS guidelines [2–5, 10–12]

*AGPs* aerosol generating procedures, *BLS* basic life support, *BVM* bag valve mask, *COVID-19* coronavirus disease 2019, *CPR* cardiopulmonary resuscitation, *HEPA* high-efficiency-particulate-air, *PPE* personal protective equipment, *SARS-CoV-2* severe acute respiratory syndrome coronavirus-2

**Table 2:** Do’s and Don’ts of the COVID-19 adult BLS guideline protocols for healthcare providers

Do’s of the COVID-19 adult BLS guideline protocols to reduce exposures to SARS-CoV-2	Don’ts of the COVID-19 adult BLS guideline protocols to avoid protocol deviations and errors
Assume an OHCA patient with suspected COVID-19 [2, 3]	Start CPR without donning appropriate transmission-based precautionary PPE [10]
Practice hand hygiene [17] before donning appropriate PPE	Use an ineffective face shield, pocket mask [10, 11], or an unfit transmission-based mask and/or unsealed goggle
Use minimum airborne-precaution PPE or droplet-precaution PPE [10] if unavailable	Delay in early defibrillation [11] due to AED retrieval delay or changing an old battery with poor functional quality
Cover the patient’s mouth and nose with a fluid-resistant surgical mask or oxygen mask if available [3, 10–12]	Check and attempt to clear the patient’s airway [3] in proximity and/or touch the patient’s body fluids or discharge
Check for response; if unresponsive, use mobile phones to send helper(s) or shout for nearby help to activate EMS and to fetch an AED [2, 12] or BLS-related equipment if available	Listen and feel near the patient’s mouth to check for breathing [3] in proximity
When no cervical spine injury is suspected, open the airway with a head-tilt/chin-lift [3] maneuver	Perform mouth-to-mouth or mouth-to-nose rescue breathing [10]
Check the breathing and the pulse simultaneously [2, 12]. Look for no breathing or only gasping, and palpate for thoracic respiratory movements [2]	Provide positive pressure ventilation without wearing appropriate PPE [3]
Prioritize defibrillation before chest compression [2, 3, 10, 11]	Perform BVM ventilation without a HEPA filter between the mask and the bag [10]
Prioritize oxygenation and ventilation [2, 10] if available and trained, and use BVM ventilation with a HEPA filter to provide rescue breathing if available and with two rescuers	Perform BVM ventilation by a single rescuer with a single hand holding the mask, resulting in inadequate sealing, and another hand squeezing the bag [3]
Use a mechanical chest compression device if available and trained [2, 3, 11, 12]	Perform chest compressions during BVM ventilation [3, 10, 11]
Every 2 min, rotate the rescuer, providing a 30 chest compressions/2 rescue breathing cycle [23, 24] if there are two rescuers [11], ideally during the rhythm and pulse check	Continue chest compressions if exhausted or if the rescuer develops “CPR-induced over-performing syndromes,” such as shortness of breath, hyperventilation, and chest pain [23]
Use the two-handed, two-person BVM technique: one rescuer holds the BVM with two hands to ensure a good seal, and the second rescuer squeezes the bag twice for rescue breathing when pausing after each 30 chest compressions [2, 10]	Touch the mucosae of the eye, nose, and mouth with a hand during the doffing of used PPE [40]
Doff all the used PPE in the appropriate sequence and apply hand hygiene after CPR [3]	Discard used disposable PPE in an open bin without disinfection
Clean and disinfect appropriately all the used BLS-related equipment [3]	Forget to clean and disinfect the environment [16], such as the used BLS-related equipment, the bio-waste bin, and/or the surfaces and air in the CPR room

Compiled and modified based on COVID-19-specific BLS guidelines and literature [2, 3, 10–12, 16, 17, 23, 24, 40]

*AED* automated external defibrillator, *BLS* basic life support, *BVM* bag valve mask, *CPR* cardiopulmonary resuscitation, *COVID-19* coronavirus disease 2019, *EMS* emergency medical service, *HEPA* high-efficiency-particulate-air, *OHCA* out-of-hospital cardiac arrest, *PPE* personal protective equipment, *SARS-CoV-2* severe acute respiratory syndrome coronavirus-2

## Current evidence of CPR-associated SARS-CoV-2 aerosol transmission

There is increasing evidence of the possible risk of CPR-associated SARS-CoV-2 aerosol transmission. A recent systematic review found indirect evidence that CPR was associated with aerosol generation and the transmission of infection but was unsure whether chest compression or defibrillation caused bioaerosols or the transmission of COVID-19 to rescuers [13]. Another systematic review concluded that there was sufficient growing evidence of agreement across different international guidelines to classify certain resuscitation procedures, such as intubation, CPR, and manual ventilation, as AGPs [14]. In early 2020, the WHO categorized CPR as an AGP [15, 16]. There is insufficient evidence to suggest that chest compression is not an AGP with a risk of transmission [17]. Bio-aerosol nuclei may remain in the air of a poorly ventilated room for a longer time. Notably, viable SARS-CoV-2 can persist in aerosols for 3 h [18]. A recent simulation and cadaver study found that most aerosols spread in the direction of the provider during chest compression-only CPR without any airway device and that using a surgical mask or an oxygen mask as a barrier device on the patient’s face would reduce the aerosol spread [19]. In the 2022 interim COVID-19 adult BLS update, the American Heart Association aligns with the most recent WHO and the Centers for Disease Control and Prevention; it now considers CPR and all its components (e.g., chest compressions, ventilation, and defibrillation) aerosol-generating [5].

## The COVID-19-specific adult BLS guideline strategies for chiropractors and other healthcare providers

### Precaution strategies

COVID-19 resuscitation safety is paramount, and the priorities are (1) the self, (2) the colleagues and bystanders, and (3) the patient [10]. Prehospital healthcare providers should follow the standard droplet plus aerosol precautions when resuscitating suspected or confirmed COVID-19 patients [2, 3, 5, 10, 11]. In determining the appropriateness of resuscitation, it is paramount to consider the severity of the illness and the rescuer’s age and comorbidities [2].

There was disagreement among the COVID-19 BLS guidelines on the level of PPE. A precautionary approach with the bare minimum of appropriate PPE for AGPs is recommended to protect healthcare providers from contracting SARS-CoV-2 in suspected COVID-19 patients with BLS-associated AGPs [12, 17]. Cautiously, CPR should not be started on any patient with suspected or confirmed COVID-19 until the team is fully attired with appropriate PPE [11] against both airborne and droplet particles [2]. All healthcare rescuers should use airborne-precaution PPE like a respirator of the European Union Standard filtering facepiece (FFP) 2, FFP3, or the United States National Institute for Occupational Safety and Health-certified N95 [2, 6, 10] along with other PPE (gown, gloves, and eye protection) for AGPs with suspected or confirmed COVID-19 patients [6].

### Resuscitation strategies

#### Prioritizing defibrillation

In the chain of survival [20, 21], prioritizing rapid defibrillation before chest compressions [2–4, 10, 11] and attention to reversible causes of cardiac arrest remain the critically modified interventions [3] during the COVID-19 pandemic. Because the early return of spontaneous circulation (ROSC) may negate the need for chest compressions and ventilation, which are classified as AGPs [14], or the need for invasive airway management and invasive ventilation. Notably, different from other COVID-19 BLS guidelines, the International Liaison Committee on Resuscitation and the European Resuscitation Council suggested defibrillation before donning airborne-precaution PPE for AGPs in situations where the benefits might exceed the risks [4, 10], because of the potential for defibrillation within the first few minutes to achieve a sustained ROSC and the unlikelihood of defibrillation generating an aerosol [4, 10].

#### Prioritizing oxygenation and ventilation

Supplementing the chain of survival during the COVID-19 pandemic and the benefit of supplemental oxygen during CPR remain uncertain and debatable [22, 23]. However, ROSC cannot be achieved without re-oxygenating the ischemic myocardium following cardiac arrest [23]. Hence, for agonal breathing, consider passive oxygenation overlaid with a surgical mask (if available) [5, 12] when a BVM with a high-efficiency-particulate-air filter is not being utilized [5, 12]. As a result, during chest compression-only CPR [2], oxygenation and ventilation should be prioritized as soon as possible [10]. Vigilantly avoid or minimize positive-pressure ventilation by a BVM. Two rescuers should be present if required to allow a two-handed, tight seal [3, 11]. Preferably, using a BVM with a high-efficiency-particulate-air filter before chest compressions, if available, will minimize exposure to SARS-CoV-2 [5, 10–12].

#### Emphasizing the high quality of chest compression-only CPR

Chest compression is a critical component of effective CPR to perfuse vital organs during cardiac arrest. Importantly, initiate chest compressions immediately in the event of a witnessed sudden cardiac arrest, such as a possible exercise-related OHCA in chiropractic settings. If not already masked, healthcare providers should don their masks without delay or interruption with chest compressions [12]. The COVID-19 adult BLS guidelines [3, 11] also emphasize the concept of high-quality CPR as one of the significant parts of the “chain of survival” [20, 21]. Effective, timely, high-quality manual chest compression-only CPR [3, 11] is an essential element of managing an OHCA to achieve a successful ROSC during the COVID-19 pandemic.

#### Optimizing chest compression rate, depth, recoil, and fraction

Previous studies suggested that the quality of chest compression delivered was mainly associated with chest compression rate, depth, recoil, and fraction [20, 21, 24]. Chest compression should have the following parameters [11, 23–25]: chest compression *rates* between 100 and 120 per minute and a chest compression *depth* of about 5–6 cm. Chest compression rates between 100 and 120 per minute [25] were associated with the greatest survival following an OHCA and hospital discharge [26]. The optimal chest compression velocity with full *recoil* is associated with a significant improvement in survival and a favorable neurological outcome at hospital discharge after an OHCA [27]. A chest compression *fraction* (CCF) is the proportion of time spent providing chest compressions while the patient is pulseless and in cardiac arrest. Previous observational studies found that shorter pauses [28] or higher CCF were associated with higher odds of survival to hospital discharge for OHCA patients [29, 30]. According to the 2021 and 2022 interim COVID-19 adult BLS guidelines, maximize CCF and pause to intubate only if needed [5, 12].

#### Attenuating the impact of wearing PPE on the quality of manual chest compression-only CPR

The impact of wearing PPE during the COVID-19 pandemic on the quality of manual chest compression-only CPR was left unaddressed in the 2020 COVID-19 BLS guidelines [2–4, 10, 11], except for the updates in the 2021 and 2022 COVID-19 BLS guidelines that PPE may have accelerated rescuer fatigue, resulting in decreased CPR quality [5, 12]. Recent studies found a significant increase in adverse reactions such as PPE-induced fatigue [31–34], thermal discomfort, dizziness, and heat stress [35] among healthcare providers during CPR. Also, a recent systematic review and meta-analysis of the impact of PPE on the effectiveness of chest compression concluded that the use of PPE compromised the quality of chest compression during CPR significantly [36]. Cooling strategies to attenuate PPE-induced heat strain [37], such as wearing a cooling vest [37] before, during, and after CPR during the COVID-19 pandemic, should be considered, implemented, and followed.

### Education strategies

Like other healthcare providers, infectious disease transmission during chiropractic CPR training is a common concern [38]. The European Resuscitation Council highlighted the importance of sustaining BLS and provided interim guidelines for resuscitation education strategies to minimize the risk of infection during the COVID-19 pandemic [10].

#### Transitioning to the COVID-19 BLS web-based e-education and e-training

Like telehealth education and training, specific web-based BLS e-education and e-training require an innovative, modified approach to the traditional training during the COVID-19 pandemic to host “tele-BLS” courses. All healthcare professionals with a duty of care to respond but rarely treat OHCA patients should also undergo self-directed BLS e-learning and virtual skill e-training [10], including donning and doffing PPE [11, 37]. During e-training, potential self-contamination during the doffing of used PPE [39, 40] due to mucosal exposure of the eye, nose, and mouth by touching the face with the hand(s) should be warned. Also, an appropriate proper doffing-PPE sequence should be demonstrated, practiced, and evaluated with feedback. Many web-based BLS courses and COVID-19-related infection prevention and control courses are available for healthcare providers. Samples of the free WHO web-based COVID-19-relevant courses [41] are:

- “Infection prevention and control in the context of COVID-19,” available at https://openwho.org/courses/COVID-19-IPC-EN.
- “COVID-19: How to put on and remove personal protective equipment,” available at https://openwho.org/courses/IPC-PPE-EN.

## Conclusions

This commentary is the first article on the current COVID-19-specific adult BLS guidelines for chiropractors to maximize the safety and efficacy of resuscitation. It has relevance and impacts future research in areas such as SARS-CoV-2 infection prevention and control strategies. It provides a practical overview and highlights the current intervention strategies of the COVID-19 interim adult BLS guidelines that may help chiropractors and other healthcare providers reduce BLS-related exposures to SARS-CoV-2 and the risks of SARS-CoV-2 transmission and maximize the efficacy of CPR. Chiropractors and other healthcare providers should follow current COVID-19-specific intervention strategies during the pandemic. Future studies should focus on evaluating the effectiveness of COVID-19-specific interventions in adult BLS guidelines, although studies to assess the effectiveness of interventions may not be ethically feasible.

## Data Availability

Not applicable.

## Abbreviations

AGP: Aerosol-generating procedure
BLS: Basic life support
BVM: Bag-value mask
CCF: Chest compression fraction
CPR: Cardiopulmonary resuscitation
FFP: Filtering face piece
HEPA: High-efficiency-particulate-air
OHCA: Out-of-hospital cardiac arrest
PPE: Personal protective equipment
ROSC: Return of spontaneous circulation
SARS-CoV-2: Severe acute respiratory syndrome coronavirus-2
WHO: World Health Organization
